# Understanding Fibrous Tissue in the Effective Healing of Rotator Cuff Injury

**DOI:** 10.26502/jsr.10020363

**Published:** 2024-05-21

**Authors:** Resmi Rajalekshmi, Devendra K. Agrawal

**Affiliations:** Department of Translational Research, College of the Osteopathic Medicine of the Pacific, Western University of Health Sciences, Pomona, California USA

**Keywords:** Fibrous tissue, Rotator cuff, Collagen, Pathways, Tendons

## Abstract

The rotator cuff is a crucial group of muscles and tendons in the shoulder complex that plays a significant role in the stabilization of the glenohumeral joint and enabling a wide range of motion. Rotator cuff tendon tears can occur due to sudden injuries or degenerative processes that develop gradually over time, whether they are partial or full thickness. These injuries are common causes of shoulder pain and functional impairment, and their complex nature highlights the essential role of the rotator cuff in shoulder function. Scar formation is a crucial aspect of the healing process initiated following a rotator cuff tendon tear, but excessive fibrous tissue development can potentially lead to stiffness, discomfort, and movement limitations. Age is a critical risk factor, with the prevalence of these tears increasing among older individuals. This comprehensive review aims to delve deeper into the anatomy and injury mechanisms of the rotator cuff. Furthermore, it will inspect the signaling pathways involved in fibrous tissue development, evaluate the various factors affecting the healing environment, and discuss proactive measures aimed at reducing excessive fibrous tissue formation. Lastly, this review identifed gaps within existing knowledge to advance methods for better management of rotator cuff tendon injuries.

## Introduction

The rotator cuff, as an indispensable pillar of the shoulder complex, plays a pivotal role in ensuring joint stability and enabling an impressive degree of motion. Consequently, injuries to this essential assembly of muscles and tendons, particularly tendon tears, have the potential to significantly disrupt an individual’s daily activities and overall quality of life. The majority of rotator cuff tendon tears are usually classified as either partial-thickness or full-thickness lesions. Notably, such injuries can either be classified as acute, occurring because of an unexpected trauma, or chronic, which may develop progressively due to repetitive or continuous stress, or degenerative alterations within the tendon substrate. Age stands out as a vital risk factor, given the rising prevalence of these tears amongst older individuals - a fact that underscores the degenerative essence of these conditions. Fibrosis constitutes a fundamental aspect of the healing process initiated following a rotator cuff tendon tear. The dual role of fibrous tissue in the healing process is significant yet paradoxical. While playing an essential role in the recovery of damaged tissues, overproduction can lead to stiffness, discomfort, movement limitations, and impede the restoration of normal tendon structure and optimal function. This dual nature of fibrous tissue requires a careful balance to achieve optimal restoration of affected tissues without causing discomfort or movement limitations.

Our research aims to provide a detailed understanding of the biological processes and mechanisms involved in the development of fibrous tissue, and its crucial role in the restoration of tendons. Our primary objective is to shed light on the various classifications of rotator cuff tears, the distinct phases of natural healing, the complex signaling pathways responsible for fibrous tissue development, and the multitude of internal and external factors that influence the healing environment. We also aim to evaluate the impact of different treatment methods on fibrous tissue growth and explore proactive measures that can reduce excessive fibrosis. Our goal is to identify potential areas for further research and propose new strategies that can enhance the management of rotator cuff tendon injuries.

### Anatomy and injury mechanisms of the rotator cuff tear

#### Anatomy

**Figure 1:**
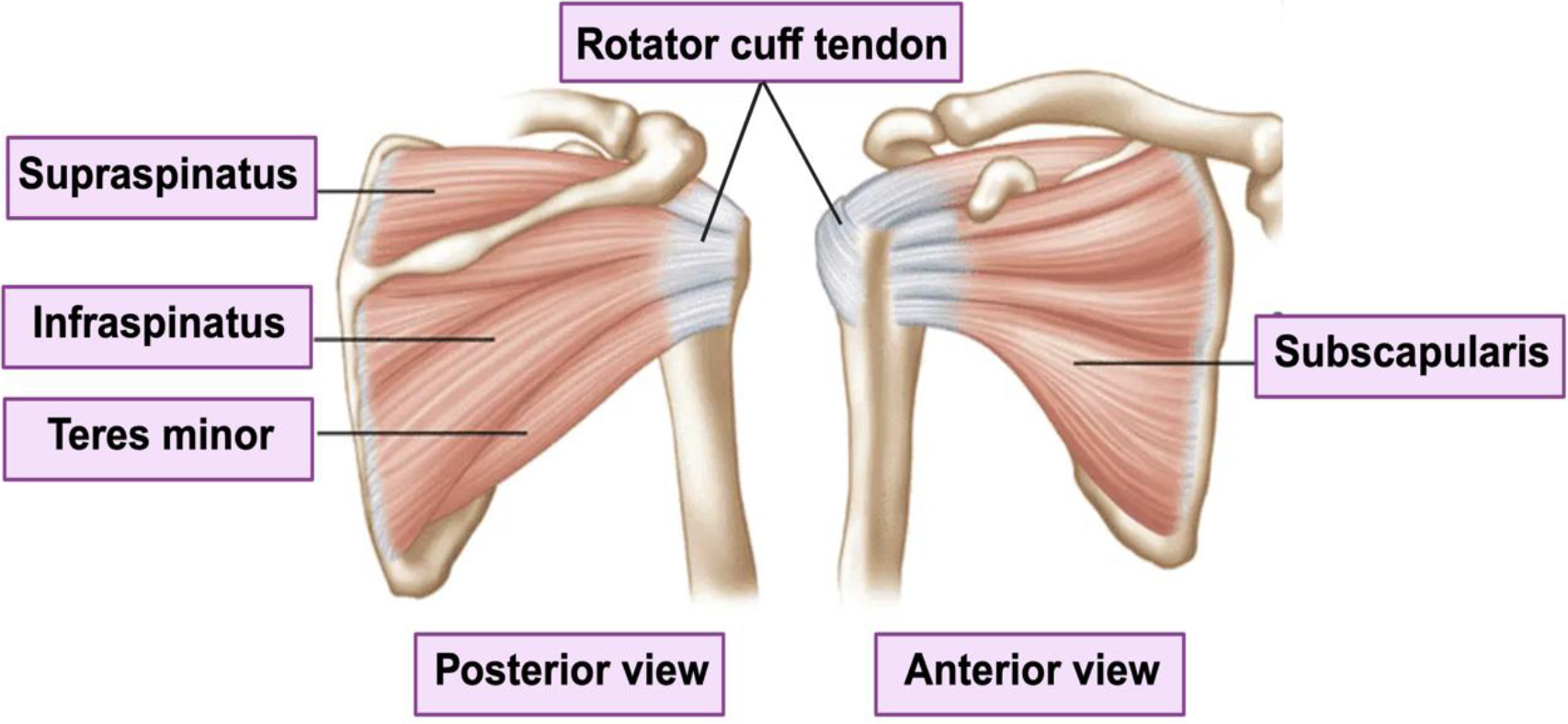
Anatomy of rotator cuff showing tendon and muscles

The rotator cuff, comprised of the supraspinatus, infraspinatus, teres minor, and subscapularis muscles (SITS), forms a vital musculotendinous complex crucial for stabilizing the glenohumeral joint and optimizing shoulder function during upper extremity movements. This acronym, SITS, succinctly designates these integral components (Figure 1) [1]. The supraspinatus, originating superior to the scapular spine, inserts on the greater tuberosity of the humerus [2], while the infraspinatus, originating below the scapular spine in the infraspinatus fossa, inserts on the posterior aspect of the greater tuberosity [3]. The teres minor, originating from the lateral border of the scapula, inserts on the inferior aspect of the greater tuberosity, distinguishing it from the non-rotator cuff muscle, teres major [4]. The subscapularis muscle, originating on the anterior surface of the scapula directly over the ribs, inserts on the lesser tuberosity of the humerus [5]. Together, these muscles intricately coordinate to precisely control the direction, degree, and quality of humeral head motion, playing a pivotal role in ensuring optimal shoulder functionality and preventing pathology associated with the complex biomechanics of the shoulder joint.

### 1.1. Rotator cuff injuries

Rotator cuff injuries, a prevalent source of shoulder pain and functional impairment, impact the tendons and muscles integral to stabilizing the shoulder joint and enabling diverse arm movements. These injuries, stemming from acute trauma, overuse, or degenerative processes, encompass various types.

Rotator cuff tendinitis, marked by tendon inflammation, commonly arises from overuse, repetitive arm motions, or the natural ageing process [6]. Rotator cuff tears, whether partial or complete, result from acute injuries or degenerative changes [7]. Impingement syndrome, characterized by the pinching of rotator cuff tendons between the acromion and humeral head, induces pain and restricts movement [8]. Concurrently, rotator cuff bursitis, an inflammation of the bursa between the rotator cuff and acromion, often accompanies tendinitis [9]. Calcific tendinitis involves the deposition of calcium within rotator cuff ten dons, culminating in pain and restricted range of motion [10]. These diverse injuries collectively underscore the pivotal role of the rotator cuff in shoulder function and the multifactorial nature of the conditions afflicting this critical musculoskeletal structure.

#### Rotator cuff tears

A rotator cuff tear arises from the rupture of one or more tendons within the rotator cuff, a group of muscles and their associated tendons in the shoulder. Etiologically, such tears can be attributed to either acute traumatic incidents or degenerative processes occurring gradually over time. The systematic classification of rotator cuff tendon injuries plays a critical role in various aspects, including accurate diagnosis, planning suitable treatments, and helping forecast the possible progress and outcomes of the condition.

**Figure 2:**
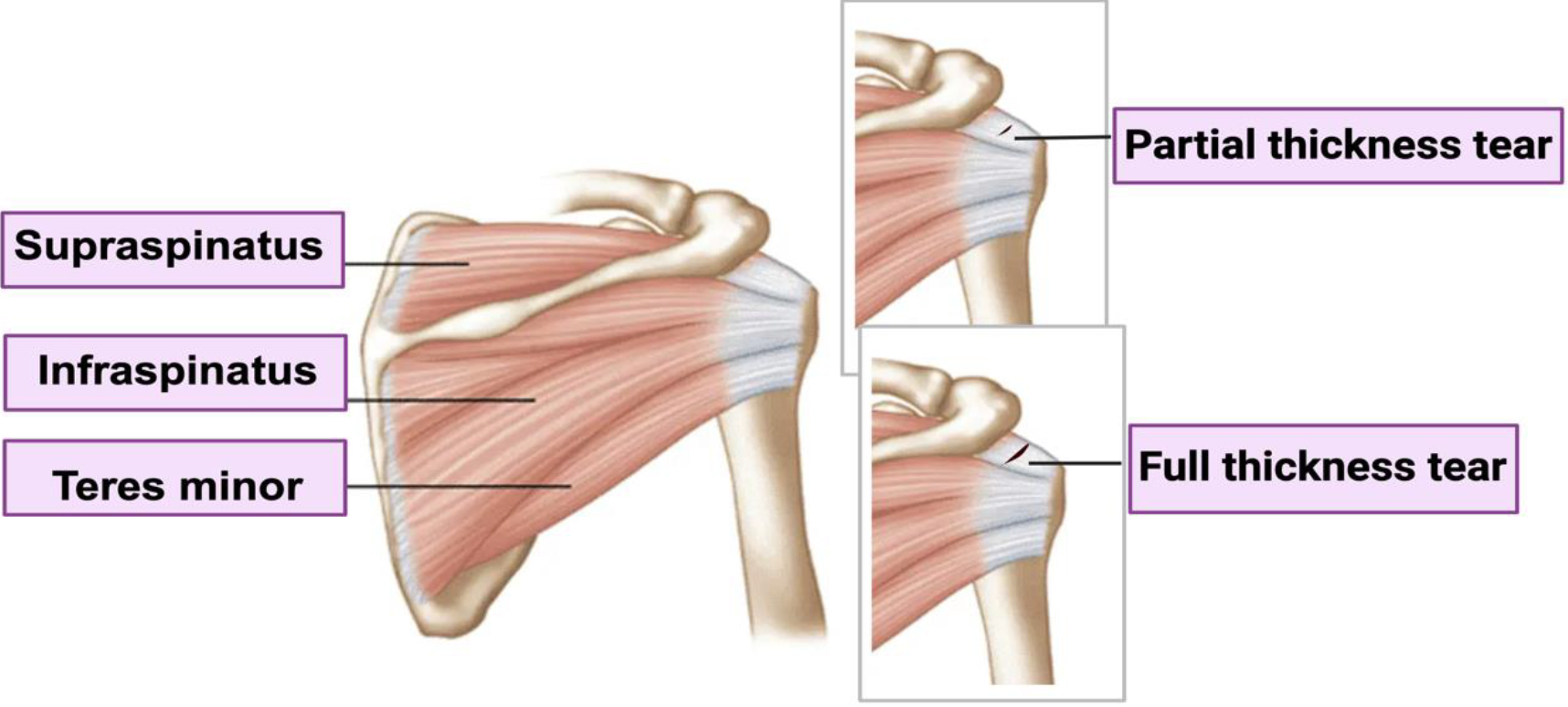
Rotator cuff tears in tendons

Rotator cuff tendon injuries are traditionally bifurcated into two core categories according to their severity - these are partial-thickness and full-thickness tears as shown in figure 2 [11,12]. Partial-thickness tears, sometimes referred to as incomplete tears, involve injury to only part of the tendon [13]. On the other hand, full-thickness tears are a more severe type of injury that involves damage traversing the entire thickness of the tendon [14]. This culminates in a complete discontinuity between the tendon and its insertion points on the humerus. The therapeutic challenge posed by full-thickness tears is elevated due to possible complications, such as muscle atrophy, tendon retraction and fatty infiltration that can exacerbate the complexity of repair if left untreated [15]. The percentage of partial-thickness tears is higher (18.5%) than that of full-thickness tears (11.7%) [16]. Among those 20 years of age and younger, 8.7% have rotator cuff abnormalities, while the prevalence increases to 62% with age [17].

Manifesting clinically, individuals with rotator cuff tears typically present with symptoms encompassing shoulder pain, muscular weakness, and constrained range of motion. These clinical manifestations collectively characterize the deleterious impact of rotator cuff tears on the shoulder’s structural and functional integrity.

#### Pathophysiology of fibrous tissue formation

The fibrosis is intimately associated with an aberrant microenvironment characterized by dysregulated molecular and cellular events. During normal wound healing, the tissue undergoes a precisely orchestrated sequence of events aimed at restoring structural integrity. However, during fibrosis, this equilibrium is disrupted, leading to persistent alterations in the local microenvironment.

In the context of rotator cuff tendon injury, understanding the pathophysiology behind scar tissue formation provides crucial insights for clinicians, physical therapists, and patients alike. When a tendon experiences damage, the exact process of healing executed by our body does not always expertly mimic the natural architecture of the tissue, often resulting in the formation of fibrous tissue.This reparative tissue is seen as a vital component in the early phase of tendon recovery; however, its continued maturation and development can introduce complications in the healing pathway, potentially influencing the overall function of the tendon and impacting the outcome of the recovery process [18].

Beyond just the immediate implications for healing, an in-depth exploration of these pathophysiological processes promises insights into the broader field. A deeper understanding of the molecules and cells involved, the stages of healing, and the numerous factors that can influence the quality of the results will help in developing targeted therapies and optimizing rehabilitation protocols. If we can learn to modulate these processes effectively, we can improve the outcome following a tendon injury and minimize the long-term detriments that often accompany this type of impairment.

### 1.2. Abnormal cells involved in fibrosis

Intrinsic healing is driven by cells derived from the tendon parenchyma and epitenon/endotenon, whereas extrinsic healing is attributed to cells recruited into the injury area from outside the tendon itself (e.g. circulating cells, cells from neighboring tissues including the paratenon and sheath, etc.). Both sources of cells are important, however, conditions that promote increased contribution from extrinsic cell populations are thought to result in greater fibrous tissue and adhesion formation compared to those that promote intrinsic healing. The importance of understanding the activity of intrinsic vs. extrinsic cells in tendon healing is highlighted by studies of tendon healing during embryonic development. The difference between the two healing paradigms is thought to be due to decreased contribution from extrinsic sources (inflammatory cells, paratenon/sheath cells, bone marrow-derived cells, etc.) and increased activity from intrinsic tendon cells in the embryo.

When adult tendons are injured, they heal by forming a mechanically inferior, fibrous tissue. However, fetal tendons heal by regeneration, which is a scar-less, more regenerative healing process that is also conserved during the early neonatal period [19,20]. Although the overall process of tendon healing is relatively well understood, there is still less knowledge about the identity and behavior of cells involved at each stage. This includes their potential contributions to pathologic fibrosis.

#### Macrophages

Macrophages play a pivotal role in orchestrating wound repair by providing essential signaling molecules to coordinate the intricate process of wound healing. Activated macrophages exhibit two distinct subtypes: the M1 subtype, associated with inflammatory responses, and the M2 subtype, associated with tissue modeling and anti-inflammatory responses [21]. M1 macrophages engage in the phagocytosis of pathogens and cellular debris, secrete pro-inflammatory factors, and contribute to the inflammatory phase of wound healing [21]. Conversely, M2 macrophages predominantly participate in the proliferation and remodelling phases, actively contributing to granulation tissue formation, remodeling processes, and the resolution of inflammation [22]. It is crucial to note that sustained activation of M1 macrophages can result in chronic inflammation, leading to delayed wound healing. On the other hand, excessive activation of M2 macrophages can contribute to a high rate of fibrosis and scar formation [23].

Lin et al. observed that an increased ratio of M1 to M2 macrophages was associated with heightened aberrant extracellular matrix (ECM) deposition post-injury, suggesting that M1 macrophages play a role in driving fibrosis during tendon healing [24]. Excess M2 macrophage activity and matrix synthesis contribute to the fibrotic healing observed in diabetic tendons, leading to compromised biomechanical strength compared to repaired tendons in the nondiabetic control group [25]. The excessive fibrosis in these cases may be attributed, in part, to the overproduction of TGF-β1 by M2 macrophages, a factor linked to the development of pathological fibrotic conditions in various tissues [26].

Despite M2 macrophages demonstrating a “pro-regenerative” effect through new matrix synthesis, the resulting tissue does not regenerate the normal tendon structure, composition, and material properties.

##### 1.2.1.1. Myofibroblast

Myofibroblasts, characterized by de novo expression of α-smooth muscle actin (αSMA) and mechanosensitive stress fibers, serve as key regulators in both physiological wound healing and the development of tissue fibrosis [27]. The precise origins of myofibroblasts in the context of wound healing remain uncertain. However, it is hypothesized that several mechanisms contribute to their emergence. These mechanisms include the activation of resident fibroblasts or pericytes through the influence of pro-inflammatory cytokines such as TGF-β1 and interleukin-1β (IL-1β), the transformation of epithelial cells via a phenomenon known as epithelial-to-mesenchymal transition, and the recruitment of circulating cells derived from the bone marrow to the site of injury [28]. These cells play a pivotal role in extracellular matrix (ECM) production and exhibit inherent contractile abilities that modulate the stiffness of the wound matrix, influencing the behavior of other embedded cells [29]. While myofibroblasts are essential for proper wound healing through ECM synthesis and remodeling, their inappropriate accumulation or persistence at the injury site is a major factor triggering fibrotic progression [30]. This dysregulation can lead to a pro-fibrotic feedback loop, as myofibroblasts can induce the release of latent transforming growth factor-beta (TGF-β) from the ECM, and TGF-β promotes further myofibroblast differentiation [31,32]. Moreover, the physiological resolution of healing is hindered by the inadequate clearance of myofibroblasts, either through apoptosis or reversion to their basal fibroblastic state.

##### 1.2.1.2. Scx-lineage (Scx^Lin^) cells

Scleraxis-lineage cells are involved in tendon homeostasis, maintenance, and response to injury. They contribute to the production and organization of collagen fibers, which are essential for tendon strength and function [33].

Research studies employing models such as the patellar window defect [33] and partial Achilles transection [34] have substantiated the localization of Scleraxis-lineage cells within the resultant scar tissue.

A study conducted by Best et.al; established a correlation between Scleraxix (Scx) and 100 calcium-binding protein A4 (S100A4) expression patterns in both homeostasis and the healing process. The findings revealed that adult ScxLin cells was present in the organized bridging tissue, while S100a4 cells exhibited localization throughout the entire fibrous tissue [35]. Subsequent research by the same group revealed that depleting ScxLin cells led to enhanced biomechanical properties without compromising gliding function 28 days after repair, suggesting a regenerative outcome [36].

Another research investigation led by Ackerman et.al and colleagues demonstrated that Adult ScxAi9 cells exhibit a gradual and spatially influenced activation, leading to myofibroblast differentiation. This process is instrumental in orchestrating both the elaboration of the ECM and the development of fibrosis [31].

#### Molecular Signaling Pathways in Fibrous Tissue Development

**Figure 3:**
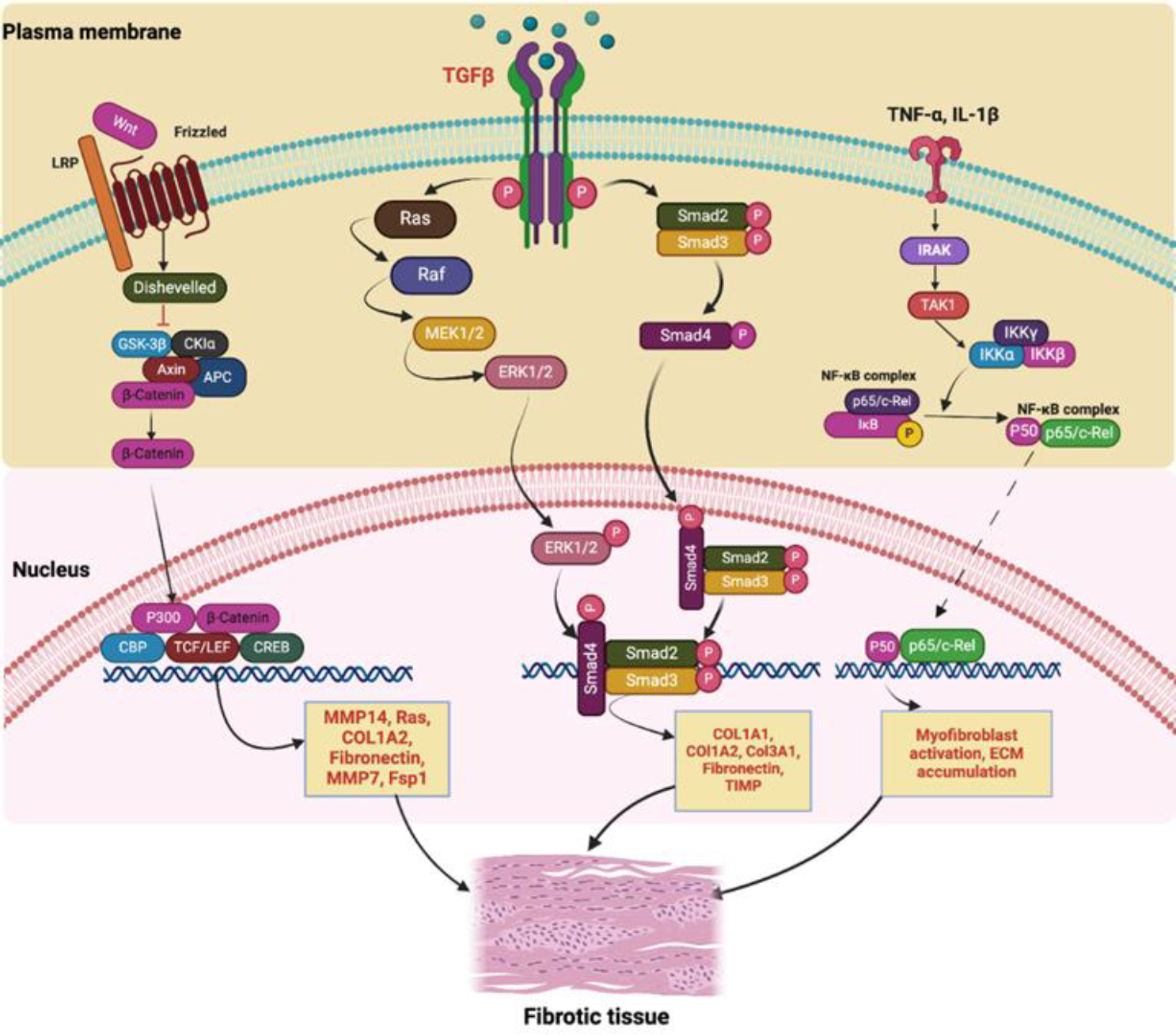
Signaling pathway showing the role of intracellular molecules and kinases in upregulating genes and proteins involved in the formation of fibrous tissue.

Fibrous tissue development holds pivotal importance in the complex process of bodily repair that ensues after injuries such as rotator cuff tendon tears. This key phase is orchestrated through a sophisticated network of molecular signaling pathways, which serve as a map guiding the reparative process from initiation to resolution. These signaling pathways do not act in isolation but rather are part of a comprehensive, interconnected system that involves the active participation of a range of chemical and biological mediators. Each of these functions in a definitive capacity, playing an instrumental role in the successive stages of inflammation, cell proliferation, and ultimately, the equilibrium poised between regenerative healing and fibrotic healing activities (Figure 3).

As soon as an injury occurs, the body’s immediate response is one of inflammation, which, contrary to common perception, is a complex process set in motion for tissue repair. This initial response sets off a cascade of molecular events, part of an intrinsic, programmed response of the body to any form of tissue damage. Pro-inflammatory cytokines secreted by M1 macrophages such as Interleukin 1 (IL-1) and Tumor Necrosis Factor Alpha (TNF-α), serve a dual purpose in this process [37]. Not only do these molecules signal immune cells to the injury site, thus initiating the healing process, they also activate fibroblasts - cells that synthesize and secrete collagen, playing a foundational role in wound recovery.

Inflammatory processes have a close relationship with the nuclear factor kappa-B (NF-κB) signaling transduction pathway. In the context of tendon injuries, the classic NF-κB signaling pathway is implicated in both acute and chronic scenarios. Research findings indicate the activation of the classic NF-κB signaling pathway in models of acute flexor tendon injury and repair. This activation correlates with an up-regulation in the expression of Col1a1 and Col3a1, accompanied by an increase in myofibroblasts and macrophages. [38,39]. Recent reports indicate sustained activation of canonical NF-κB signals in tendons and myofibroblasts throughout the healing phase post-inflammation, with persistent NF-κB activation observed in human tendon scar tissue. This continuous NF-κB signaling during tendon healing signifies its role in promoting the progression of tendon fibrosis [40,41]. Consequently, targeting the NF-κB signaling pathway emerges as a potential strategy to mitigate tendon fibrosis and prevent scar formation.

Upon tendon injury, the activation of transforming growth factor-beta (TGF-β) plays a pivotal role, serving as a crucial mediator in both tendon healing and the onset of tendon fibrosis [42]. TGF-β exerts a wide range of actions on fibroblasts, modulating proliferation, migration, survival, and gene expression. These actions have pronounced effects on ECM remodeling. TGF-ꞵ acts as a key modulator of fibroblast phenotype and function, inducing the transformation of fibroblasts into myofibroblasts, which are responsible for excessive extracellular matrix production and tissue scarring [43]. In this stage, TGF-β facilitates tendon fibroblast proliferation while concurrently inhibiting fibroblast apoptosis through the TGF-β/Smad3 pathway, thereby promoting effective tendon healing [44].

Upon stimulation by TGF-β, these contractile fibers undergo a transformation marked by the incorporation of α-smooth muscle actin, recognized as the predominant marker denoting the mature myofibroblast phenotype in various tissues [45,46]. In the context of fibrosis, the persistent accumulation and stiffening of scar ECM actively support TGF-β activation, further driving the differentiation and growth of myofibroblasts [47,48].

TGF-β bound to the receptor also activates ERK1/2 by sequentially activating RAS, Raf, and MEK1/2 [49]. A study conducted in a chicken flexor tendon repair model shows that the phosphorylation level of ERK in the adhesion tissue of the tendon adhesion model increased significantly in a time-dependent manner, and silencing of ERK2 by Lentiviral-Mediated RNA Interference has an inhibitory effect on tendon adhesion [50]. These indicate the role of the ERK1/2 signaling pathway in tendon fibrosis. In addition, inflammation plays an important role in promoting tendon fibrosis. Studies have shown that pro-inflammatory factors can activate the ERK1/2 signaling pathway and participate in the regulation of the TGF-β/Smad signaling pathway and down-regulate the expression of the Serpin E1 gene, leading to tendon fibrosis [51].Top of Form

When Wnt/β-catenin pathway is excessively activated, it leads to an overproduction of collagen and the formation of excessive scar tissue [52]. In tendon fibrosis, the reactivation or aberrant alterations of Wnt signaling have been implicated in the differentiation of tendon stem/progenitor cells and quiescent fibroblast into myofibroblasts, enhanceing the secretion of extracellular matrix components, and triggers fibrosis [53,54].

## 2. Treatment strategies used for rotator cuff tear repair

**Figure 4:**
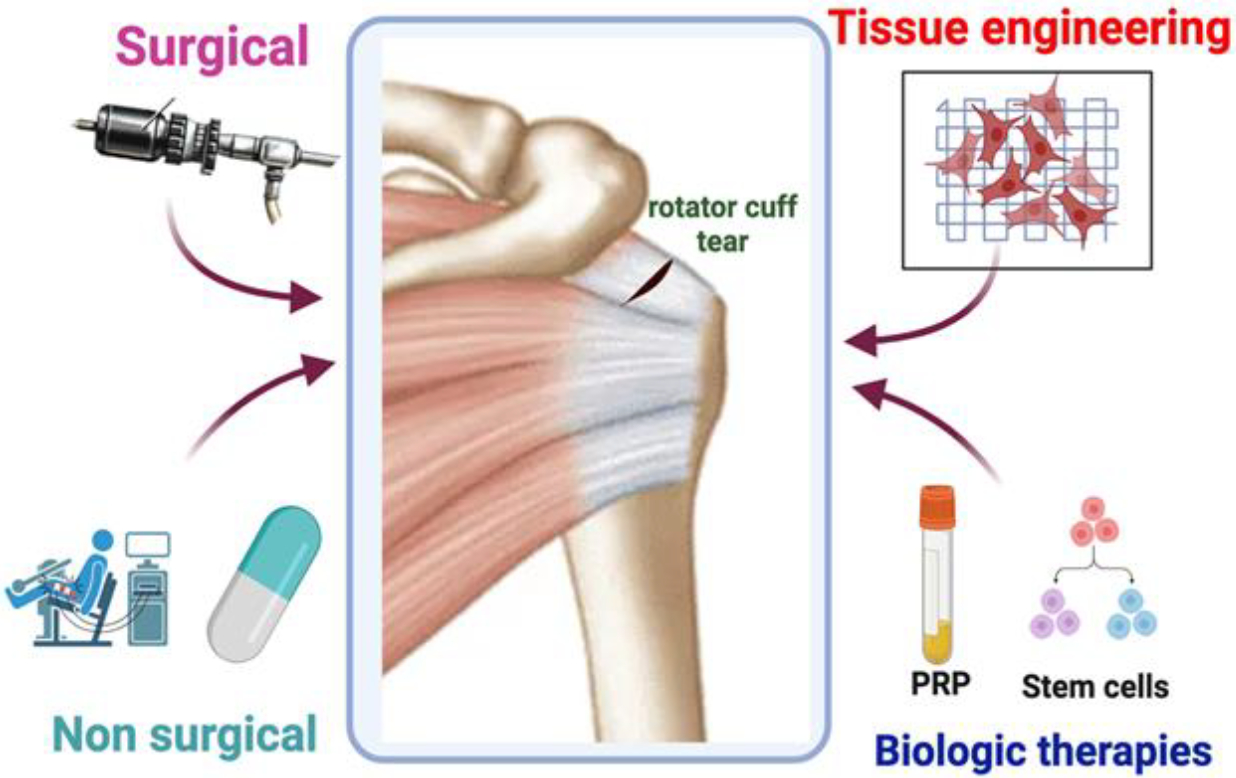
Treatment strategies using surgical and non-surgical procedures, tissue engineering, and biologic therapies using platelet-rich plasma (PRP) and stemcells for rotator cuff tear

The various treatment strategies used for rotator cuff tears was illustrated in figure 4.

### 2.1. Surgical Interventions and Associated Techniques

Rotator cuff injuries present therapeutic options encompassing conservative and surgical approaches [55]. Conservative measures involve interventions such as ice application, oral nonsteroidal anti-inflammatory drugs, and mobilization exercises. For the primary and initial management of these tears, conservative treatment stands as a reasonable option [56]. When conservative remedies fall short in producing desired outcomes, surgical repair of the damaged rotator cuff tears often comes into play. Surgical techniques ranging from arthroscopic tendon repair, traditional open surgery, or a hybrid approach involving a mini-open repair are currently in practice [57]. The continuous evolvement of surgical procedures aspires to incorporate less invasive methods, which enable quicker and less painful recovery time, simultaneously minimizing scar tissue development. Consequently, a considerable number of patients necessitate surgical intervention for tendon repair to alleviate pain and restore shoulder function following unsuccessful conservative management [58].

Surgical repair of rotator cuff tendons can be executed through either open surgery or arthroscopic-assisted procedures [59,60]. In cases of full-thickness tears, arthroscopy or mini-surgery employing a single-row technique proves effective in rotator cuff repair. On the other hand, for individuals lacking full-thickness tendon tears, arthroscopic decompression emerges as a favorable option. Recent research by Randelli et al. suggests that, for patients without full-thickness tears, surgery may not provide superior outcomes compared to conservative approaches [61].

While arthroscopic techniques have largely replaced open repairs for rotator cuff repair, both approaches demonstrate comparable long-term clinical and imaging outcomes. There is no discernible superiority of one procedure over the other, affirming the effectiveness of both in treating rotator cuff tears [62]. Statistics reveal that approximately 18–94% of patients undergoing rotator cuff surgery experience retears afterward based on the type of tear, duration of the procedure, biceps procedure, postoperative UCLA score [63] and fatty degeneration [64]. Failures in rotator cuff surgeries often necessitate costly revision procedures, including salvage methods such as reverse total shoulder arthroplasty [65].

### 2.2. Biologic Therapies

Over the past two decades, extensive studies on the biomechanics of rotator cuff repair have led to improvements in surgical techniques and repair constructs [66–68]. More recently, researchers have shifted their focus to optimizing the healing environment post-rotator cuff surgery to enhance integrity and reduce structural failure [69,70]. Biologic adjuvants such as PRP, scaffolds, and stem cells have been explored to augment healing at the tendon-to-bone interface and enhance the quality of muscle and tendon. The application of these biologics holds promise for addressing the challenges posed by large and massive rotator cuff tears, potentially contributing to improved healing outcomes and enhanced structural integrity.

#### Platelet Rich Plasma (PRP)

Existing literature comprehensively explores diverse biological adjuvants for enhancing healing, with particular emphasis on Platelet-Rich Plasma (PRP). Investigations into PRP encompass its potential benefits in both surgical augmentation and non-operative management. The PRP involves using a concentrated solution of the patient’s own blood platelets which contains high concentrations of different growth factors that have been analyzed for their capacity to stimulate healing by influencing processes such as cell migration, tissue maturation, and inflammation [71]. Top of Form

A two-year longitudinal study involving 85 patients with rotator cuff injuries, treated with dual PRP injections, suggests that PRP injection should be considered as the preferred therapeutic option for individuals with partial rotator cuff tears or inflammation, particularly in instances where previous interventions such as physical therapy and activity modification have proven unsuccessful [72].

Numerous meta-analyses examining the efficacy of PRP across varying tear sizes have yielded discordant results, demonstrating no consistent clinical superiority over untreated controls [73–75]. Subgroup analyses revealed a significant superiority of PRP treatment in patients with small and medium-sized tears compared to large and massive tears, particularly in the context of double-row repair and when PRP was applied at the tendon–bone interface in a solid formulation over a liquid one [76]. The authors proposed that this discrepancy could be attributed to reduced load on anchor points in smaller tears, coupled with better biomechanical stability and vascularization, facilitating enhanced growth factor incorporation in small to medium tears.

Despite the high retear rates following arthroscopic repair of large and massive tears, the potential for PRP to enhance healing and clinical outcomes remains an area of interest. While some randomized controlled trials limited to large and massive tears suggest PRP’s structural healing potential, their lack of extended follow-up and the inconclusive findings from meta-analyses prompt caution in recommending its use.

#### Stem cell therapies

Stem cell therapies aim to harness the healing potential of mesenchymal stem cells to enhance rotator cuff repair. Stem cell application holds significant therapeutic promise for healing. Stem cells are categorized based on their differentiation ability into embryonic stem cells (including totipotent and pluripotent) and adult stem cells (including multipotent cells such as bone marrow-derived stem cells (BMSCs), adipose-derived stem cells [ADMSC] and mesenchymal stem cells [MSCs]) [77].

Mesenchymal stem cells (MSCs) exhibit the potential to differentiate into tenocytes, chondrocytes, and osteoblasts, producing growth factors that enhance enthesis regeneration and improve repair strength and quality [78]. Studies injecting MSC secretomes into rodent models with induced massive rotator cuff tears showed a reduction in muscle fatty degeneration and atrophy, with single local injections yielding more reproducible results compared to multiple systemic injections [79]. Similar promising outcomes were observed with ADMSCs in rodent models simulating chronic subscapularis and massive tears [80,81].

Animal models have shown success in the histology and biomechanics of the BMSCs [82–84] ADMSCs [85,86], Tendon Stem/Progenitor Cells [87], Umbilical Cord-Derived Mesenchymal Stem Cells [88,89], Bursa-Derived Cells [90,91], Urine-Derived Stem Cells [92] and muscle stem cells [93], but human studies, especially in large and massive tears, remain limited.

In a double-blinded, randomized, placebo-controlled trial involving patients with partial-thickness tears of the supraspinatus tendon, the administration of Mesenchymal Stem Cell (MSC) injections demonstrated no significant impact on the improvement of pain, shoulder function, or lesion size when compared to the control group. This conclusion was drawn based on assessments that included magnetic resonance imaging (MRI) [94]. Hernigou et al. conducted a human trial comparing the 10-year outcomes of patients who underwent single-row rotator cuff repair augmented with bone marrow-derived mesenchymal stem cells (BMSCs) to a control group without augmentation, representing the longest follow-up in the literature. The study included 90 patients, and the results indicated that 87% of the BMSC group and 44% of the control group maintained intact rotator cuffs after 10 years. Notably, the investigation focused exclusively on rotator cuff tears measuring 1.5 to 2.5 cm [95].

In a separate dose-escalation trial utilizing adipose-derived stem cells (ADSCs) alone, the first human study of its kind for treating partial-thickness tears, a significant reduction in the volume of articular and bursal side defects was observed. Specifically, the mid and high-dose groups experienced an 83% and 90% decrease, respectively [96].

However, it is worth noting that no trials have been conducted to date investigating the use of ADSC augmentation in the context of full-thickness rotator cuff repairs. These findings highlight the potential efficacy of stem cell-based interventions in promoting healing and reducing defects in specific types of rotator cuff tears, while also underscoring the need for further research, particularly in the realm of full-thickness repairs. These therapies are still being studied, and their long-term effectiveness is not yet fully understood.

#### Tissue Engineering

Tissue engineering scaffolds play a critical role in providing structural support and promoting the healing process. These scaffolds can be made from various biomaterials, including natural polymers or synthetic polymers. The design of tissue engineering scaffolds for rotator cuff tendon tears often involves mimicking the native extracellular matrix (ECM) structure and incorporating growth factors or other bioactive molecules to enhance tissue regeneration. The scaffold should possess certain characteristics such as biocompatibility, biodegradability, mechanical strength, and porosity to support cell attachment, proliferation, and tissue ingrowth.

Different strategies used for preparing scaffolds include electrospinning [97–100], a 3D-bioprinting [89,101,102], hydrogels [103–106], microspheres [102,107] and decellularized matrices [108–110]. The biomaterials used in these methods include both natural and synthetic. Natural biomaterials include polysaccharides (alginate [111,112], chitosan [113,114], cellulose [115] and hyaluronic acid [116–118]) and protein (collagen [119–121], gelatin [122,123] and fibrin [124–127] biomaterials. Synthetic biomaterials include poly lactic acid (PLA) [120,128], Polycaprolactone (PCL) [129–131], Poly (lactic-co-glycolic acid) (PLGA) [132] and poly(urethanes) [133].

In terms of cell sources, researchers have explored various options for tissue engineering scaffolds in rotator cuff tendon tears. These include tenocytes [134,135], fibroblast [136,137] and stem cells [80,89,138,139]. The selected cell source should have the potential to differentiate into tenocytes, which are the main cells responsible for tendon regeneration.

Additionally, the incorporation of growth factors and other bioactive molecules into tissue engineering scaffolds can enhance the healing process. Growth factors like vascular endothelial growth factor (VEGF) platelet-derived growth factor (PDGF), transforming growth factor-beta (TGF-β), and insulin-like growth factor (IGF) can stimulate cell proliferation, collagen synthesis, and angiogenesis, facilitating tissue repair (78,116–119).

While tissue engineering scaffolds hold great promise for rotator cuff tendon tear repair, further research is needed to optimize their design, fabrication techniques, and integration with host tissues. Clinical trials and long-term studies are required to validate their efficacy and safety before widespread clinical implementation. Nonetheless, tissue engineering scaffolds present an exciting avenue for improving the treatment outcomes in patients with rotator cuff tendon tears.

#### Probing into the Success Rates and Efficacy of Diverse Treatment Techniques

Treatment efficacy for rotator cuff tears, whether it pertains to tendon healing or scar tissue reduction, is influenced greatly by various factors. These range from the nature and the severity of the tear, the timeliness of the administered treatment, patient compliance levels with assigned treatment protocols, to the suitability of the chosen treatment methodology. Although various treatments, including surgical, non-surgical and tissue engineering approaches are available for repairing rotator cuff tears, no studies to date have proven efficacy in reducing scar tissue formation rather than tendon healing. Non-surgical interventions have proven effective for many, but success rates fluctuate contingent on individual patient adherence and the specific injury characteristics. Most patients experience pain alleviation and enhanced shoulder function post-surgery; however, a notable proportion, ranging from 20% to 70%, report dissatisfaction with postoperative functional outcomes due to re-tearing and complications due to fibrous tissue [144].

Literature reports one study that investigated the effects of simvastatin on muscle function, fibrosis, and inflammation in rats with a full-thickness supraspinatus tear. Results showed that simvastatin treatment increased muscle fiber-specific force and reduced collagen accumulation and fibrosis in the muscle [145].

#### Recognized Voids in Literature and Prospective Dimensions of Future Research

There is a significant amount of research on rotator cuff tendon tears and how to effectively repair them [146–148]. However, we still have a lot to learn about this subject, especially in the context of fibrous tissue development and management. Improving our understanding of these areas is crucial to enhance treatment methodologies and improve patient outcomes.

Current academic discussions emphasize the need for a deeper understanding of the cellular and molecular processes that govern scar tissue formation and maturation. While we know that growth factors and cytokines play a role in fibrous tissue development, their precise interactions and pathways are still relatively unexplored. Additionally, the literature shows the need for a balanced ratio of COL1 and COL3 [149,150] during healing and the importance of proper mechanical loading and rest during recovery [151]. Finding the right balance is essential to prevent unnecessary scarring and the potential for further injury.

## Conclusion

The mechanisms underlying tendon degeneration and healing remain inadequately comprehended. Tendon healing is typified by the formation of fibrovascular scar tissue that fails to attain the mechanical characteristics of normal tendons, contributing to a notably high failure rate, particularly in the context of rotator cuff repairs. The fundamental mechanisms governing tendon healing, encompassing the expression of growth factors and recruitment of cell lines, are inadequately understood. Considering the limited regenerative capacity of tendons, the notion of scarless healing presents an enticing prospect. The exploration and application of mechanisms facilitating true regenerative, scarless healing in tendon tissue and its attachment sites hold promise as a potential solution to the prevailing challenge of suboptimal healing. However, substantial research endeavors are imperative to achieve this goal.
